# How High-Risk Comorbidities Co-Occur in Readmitted Patients With Hip Fracture: Big Data Visual Analytical Approach

**DOI:** 10.2196/13567

**Published:** 2020-10-26

**Authors:** Suresh K Bhavnani, Bryant Dang, Rebekah Penton, Shyam Visweswaran, Kevin E Bassler, Tianlong Chen, Mukaila Raji, Rohit Divekar, Raed Zuhour, Amol Karmarkar, Yong-Fang Kuo, Kenneth J Ottenbacher

**Affiliations:** 1 Preventive Medicine and Population Health University of Texas Medical Branch Galveston, TX United States; 2 Institute for Translational Sciences University of Texas Medical Branch Galveston, TX United States; 3 School of Nursing University of Texas Medical Branch Galveston, TX United States; 4 Department of Biomedical Informatics University of Pittsburgh Pittsburgh, PA United States; 5 Department of Physics University of Houston Houston, TX United States; 6 Division of Geriatric Medicine Department of Internal Medicine University of Texas Medical Branch Galveston, TX United States; 7 Division of Allergic Diseases Mayo Clinic Rochester, MN United States; 8 Radiation Oncology University of Texas Medical Branch Galveston, TX United States; 9 Department of Rehabilitation Sciences University of Texas Medical Branch Galveston, TX United States

**Keywords:** unplanned hospital readmission, visual analytics, bipartite networks, precision medicine

## Abstract

**Background:**

When older adult patients with hip fracture (HFx) have unplanned hospital readmissions within 30 days of discharge, it doubles their 1-year mortality, resulting in substantial personal and financial burdens. Although such unplanned readmissions are predominantly caused by reasons not related to HFx surgery, few studies have focused on how pre-existing high-risk comorbidities co-occur within and across subgroups of patients with HFx.

**Objective:**

This study aims to use a combination of supervised and unsupervised visual analytical methods to (1) obtain an integrated understanding of comorbidity risk, comorbidity co-occurrence, and patient subgroups, and (2) enable a team of clinical and methodological stakeholders to infer the processes that precipitate unplanned hospital readmission, with the goal of designing targeted interventions.

**Methods:**

We extracted a training data set consisting of 16,886 patients (8443 readmitted patients with HFx and 8443 matched controls) and a replication data set consisting of 16,222 patients (8111 readmitted patients with HFx and 8111 matched controls) from the 2010 and 2009 Medicare database, respectively. The analyses consisted of a supervised combinatorial analysis to identify and replicate combinations of comorbidities that conferred significant risk for readmission, an unsupervised bipartite network analysis to identify and replicate how high-risk comorbidity combinations co-occur across readmitted patients with HFx, and an integrated visualization and analysis of comorbidity risk, comorbidity co-occurrence, and patient subgroups to enable clinician stakeholders to infer the processes that precipitate readmission in patient subgroups and to propose targeted interventions.

**Results:**

The analyses helped to identify (1) 11 comorbidity combinations that conferred significantly higher risk (ranging from *P*<.001 to *P*=.01) for a 30-day readmission, (2) 7 biclusters of patients and comorbidities with a significant bicluster modularity (*P*<.001; Medicare=0.440; random mean 0.383 [0.002]), indicating strong heterogeneity in the comorbidity profiles of readmitted patients, and (3) inter- and intracluster risk associations, which enabled clinician stakeholders to infer the processes involved in the exacerbation of specific combinations of comorbidities leading to readmission in patient subgroups.

**Conclusions:**

The integrated analysis of risk, co-occurrence, and patient subgroups enabled the inference of processes that precipitate readmission, leading to a comorbidity exacerbation risk model for readmission after HFx. These results have direct implications for (1) the management of comorbidities targeted at high-risk subgroups of patients with the goal of pre-emptively reducing their risk of readmission and (2) the development of more accurate risk prediction models that incorporate information about patient subgroups.

## Introduction

### Background

Although it is well known that hip fractures (HFx) in older adults are a leading cause of morbidity, long-term functional impairment, and mortality [1], these outcomes are exacerbated when such patients are readmitted to the hospital within 30 days of hospital discharge after surgery, in addition to doubling their risk of 1-year mortality [2].

While many readmissions are unavoidable, unplanned hospital readmissions can easily negate the functional gains painstakingly achieved through weeks of post–acute rehabilitation and can increase the risk of infections acquired during hospital stays [3]. This loss is over and above the costs to caregivers and relatives who have to relive the stress of the original HFx episode, reorganize their work schedules to care for the patient, resulting in loss of productivity, and restart rehabilitation after discharge [3]. Across all conditions, unplanned readmissions cost almost US $17 billion annually in the United States [4], making them an ineffective use of costly resources and therefore closely scrutinized as a marker for poor quality by the Centers for Medicare & Medicaid Services (CMS) [5]. Consequently, the CMS instituted the Hospital Readmissions Reduction Program (HRRP) [6], which has imparted penalties on hospitals if their 30-day readmission rates exceeded the national average.

Although such incentives initially appeared to improve the readmission rates in US hospitals [7], recent reports argue that the start of the HRRP coincided with an increase in mortality among older adults [6,8]. This could have been because, as hospitals tightened their policies for readmission, many older adult patients were denied care, resulting in increased mortality. Furthermore, the decrease in readmission rates might merely reflect changes in the administrative and billing practices rather than an improvement in care [9]. These results suggest a need for more targeted research to comprehend the processes that precipitate readmission and clinical interventions that address the underlying causes of hospital readmission.

### Methods Used to Analyze the Risk of Pre-Existing Comorbidities in Hospital Readmission

As hospital readmissions in the older adult HFx population are predominantly for reasons not related to the HFx surgery [10], several studies have focused on using supervised machine learning methods to determine how pre-existing comorbidities (defined as one or more conditions or diseases co-occurring with a primary condition such as HFx) increased the risk of readmission [2,10-14]. Most of these studies have focused on using logistic regression to analyze the risk of readmission of single comorbidities. For example, a recent study using Medicare data conducted for the CMS, analyzed patients with total hip or total knee arthroplasty to construct a logistic regression model with variables including 29 comorbidities to predict readmission [14]. Although the above descriptive and predictive approaches have provided important insights into the role of comorbidities in the readmission of patients with HFx, such studies do not focus on understanding how multiple comorbidities *co-occur* within and across *subgroups* of patients, a critical step in the design of targeted interventions to reduce readmissions.

Although the *co-occurrence* of pre-existing comorbidities has not yet been analyzed in readmitted patients with HFx, it has been analyzed in other index conditions [15-18], such as chronic obstructive pulmonary disease (COPD), and in patient populations, such as in older adults [19-21]. Such studies have focused on using unsupervised machine learning methods such as clustering (eg, hierarchical and partitioning clustering), dimensional reduction methods (eg, principal component analysis), and visual analytics (eg, network visualization and analysis). These include a recent questionnaire-based study of senior Australians that compared several unsupervised clustering methods to analyze patterns of multimorbidities (2 or more co-occurring conditions or diseases irrespective of an index condition) in the population [20]. The results found frequent co-occurrences, such as high blood pressure and diabetes, across the study population. Another study used unipartite networks (where nodes represented comorbidities, and edges between pairs of comorbidities represented the frequency of co-occurrence in patients) to identify clusters of frequently co-occurring comorbidities [21].

Although these studies have revealed the feasibility and appropriateness of using unsupervised methods to analyze the co-occurrences of comorbidities, they have typically focused on a unipartite analysis (clustering of only comorbidities) of the data and therefore cannot reveal complex patterns of patient heterogeneity hidden within those co-occurrences. Furthermore, such analyses cannot reveal the nature and degree of overlap among such subgroups. Understanding the complexities in such overlapping patient subgroups and their risk for readmission has direct relevance to clinician stakeholders in inferring the underlying processes involved in precipitating readmission and for the design of targeted interventions to reduce the risk of readmission.

Therefore, we explored an approach that integrates a supervised combinatorial method with an unsupervised bipartite network to address 3 questions: (1) Which combinations of comorbidities confer high risk for readmission in patients with HFx? (2) How do high-risk comorbidities co-occur within and across subgroups of readmitted patients with HFx? (3) What is the association between comorbidity risk, comorbidity co-occurrence, and patient subgroups?

## Methods

### Overview

**Figure 1:**
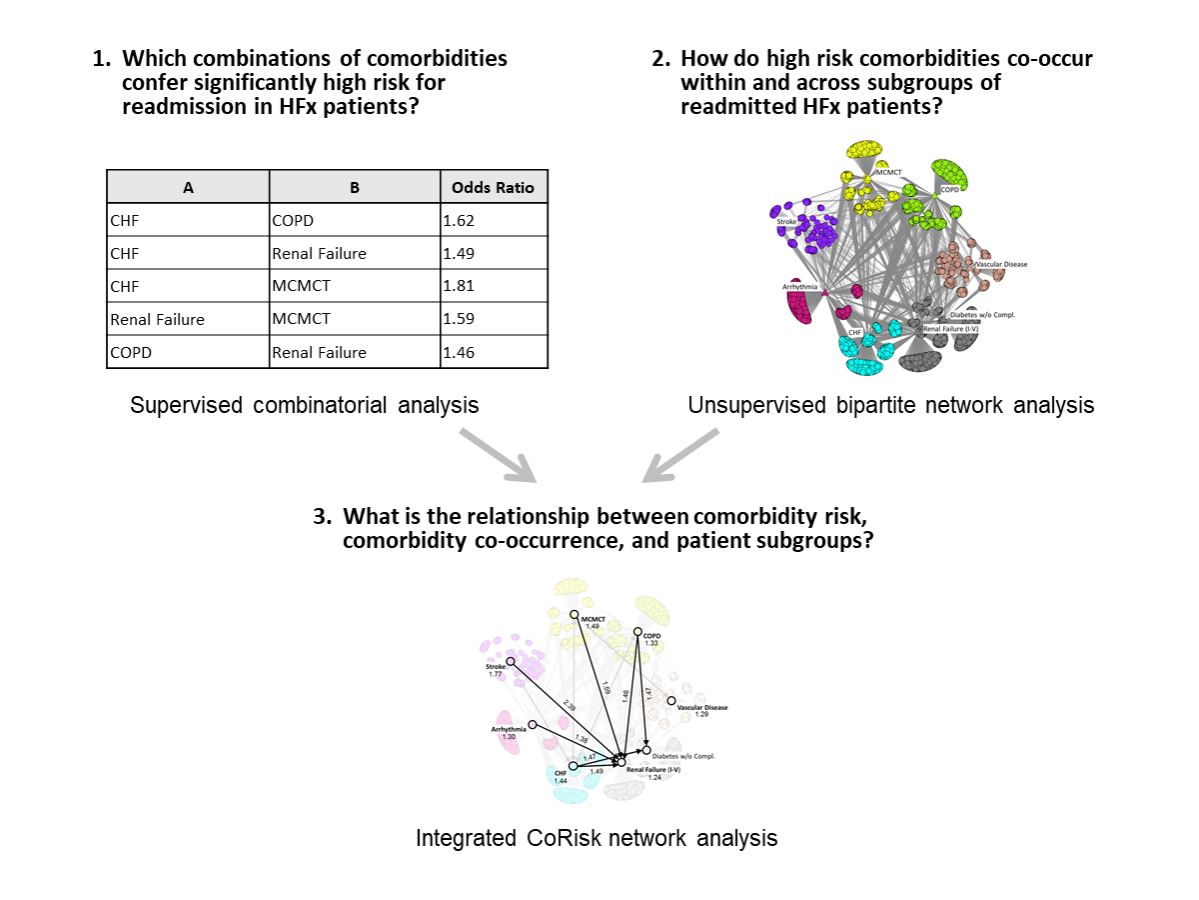
Overview of the analytical method based on 3 research questions. The steps and data shown are schematic to illustrate the overall approach and are elaborated on in the analytical method section.

As shown in Figure 1, we addressed our 3 research questions by using a supervised machine learning method to address the first question, an unsupervised visual analytical method to address the second question, and an integrated visualization of both results to address the third question. Our goal was to analyze which combinations of comorbidities confer high risk for readmission and how those high-risk comorbidities co-occur within and across patient subgroups. This integrated visual analytical approach was designed to explicitly enable clinician stakeholders using a team-centered informatics [22] approach to comprehend the complex association of comorbidity risk, comorbidity co-occurrence, and patient subgroups, with the goal of designing targeted interventions, a cornerstone of precision medicine.

### Data Selection

Our data consisted of a *training data set* extracted from the 2010 inpatient Medicare claims data (the most current Medicare data set to which we had access) and a *replication data set* extracted from the 2009 inpatient Medicare claims data (the next most current dataset). In 2010, Medicare provided health insurance to approximately 48 million Americans, of which 40 million were older adults (≥65 years), representing 93% of all older adult Americans. Furthermore, the eligible claims were from 6204 medical institutions from across the United States, thereby confirming this to be one of the few data sets that are highly representative of the US older adult population and its care.

As is commonly done in analytical studies of claims data, we used Medicare Severity-Diagnosis Related Group (MS-DRG) codes to define our population. The MS-DRG codes are used by physicians to categorize Medicare beneficiaries into payment groups for the purposes of billing. We operationally defined patients with HFx as those who were discharged from an acute care hospital with the MS-DRG codes 480, 481, or 482. To isolate the association of pre-existing comorbidities with the risk of readmission and to maintain homogeneity of our study population, we included only patients without hospital complications. Furthermore, we included only patients who were enrolled in Medicare part A but not in a health maintenance organization (a type of health insurance that limits coverage to care from contracted doctors) during the period of 90 days after discharge, in addition to patients who survived 90 days after hospital discharge.

For both the training and replication data sets, we extracted (1) the data of all patients with HFx without hospital-acquired complications who were readmitted within 30 days of discharge and (2) an equal number of controls matched for age, gender, and race, who were not readmitted within 90 days of discharge. This 90-day window of no readmittance represents an episode of care proposed by CMS for patients with HFx [23], indicating that the controls are substantially free from complications that result in readmission during this period, thereby allowing an effective comparison with the cases.

The above inclusion and exclusion criteria for patients resulted in a training data set consisting of 16,886 patients (8443 cases and 8443 controls), and a replication data set consisting of 16,222 patients (8111 cases and 8111 controls) for a total of 33,108 patients with HFx (Multimedia Appendix 1). For each of the above patients, we extracted their status on 70 high-level comorbidities (Multimedia Appendix 2) as defined by hierarchical condition categories (HCCs) [24], which represent the range of conditions typically encountered in older adults. As our index condition was HFx, we excluded it as a comorbidity, resulting in the status of 69 HCC comorbidities across 33,108 patients with HFx in the Medicare database. This retrospective study was approved by the Institutional Review Board of the University of Texas Medical Branch. The Medicare data files used for the study were in the research identifiable format, and the records were anonymized and deidentified before the analysis. Therefore, the analysis of the data did not require informed consent. Furthermore, a data use agreement was completed, which met all CMS privacy and confidentiality requirements.

### Analytical Methods Based on Research Questions

#### Which Combinations of Comorbidities Confer High Risk for Readmission in Patients With HFx?

To address this research question, we used a supervised combinatorial method to identify and replicate comorbidities that conferred high risk for readmission. Combinatorial methods have been used to analyze the prevalence of comorbidity combinations [19] and the risk of developing multimorbidities [25]. Here, we used the latter approach to identify which combinations of comorbidities confer significant risk for readmission. This analysis was performed first to base all subsequent analyses on only those comorbidities that were significant and replicated in another year.

We identified high-risk comorbidities in the training data set consisting of 16,886 patients (8443 cases and 8443 controls) by first removing all cases and controls that had none of the 69 comorbidities, resulting in 13,644 patients. Furthermore, similar to other studies on comorbidities [20], we removed 32 low-prevalence comorbidities that together occurred in less than 1% of the remaining patients (Multimedia Appendix 3), resulting in 13,512 patients in the training data set.

Next, we calculated the risk of remaining comorbidities across patients. As the patients had a median of 2 comorbidities in the HCC list, we measured the risk of all pairs of comorbidities using 2 tests. First, we used a *pairwise* *overall test * that measured the odds ratio (OR) of each pair of the 69 comorbidities compared with the rest of the patients and reported 95% confidence intervals. Second, we selected those pairs that were significant at *P*<.05 after correcting for multiple testing using the false discovery rate (FDR) method [26]. For each of the above comorbid pairs that were significant, we used a *pairwise directionality* *test* to determine the direction of their risk. Here, we conducted 2 tests: (1) A and B versus A and (2) A and B versus B, where A and B represent the sets of patients with comorbidities A and B, respectively. Within each test, we used the FDR to correct for multiple testing and considered *P*<.05 after adjustment to be significant.

To test for replication of the significance and direction of the comorbidity pairs, we repeated the above analyses using the replication data set. As the patients in the test data also had a median of 2 comorbidities, we analyzed which of the significant pairs in the training data were also significant and had the same risk direction in the replication data set. Significant comorbidity pairs that had an identical direction of risk in each data set were selected for subsequent analyses. All tests of statistical significance were two-sided, and the analyses were performed using R version 3.6.1 (R Foundation for Statistical Computing; Multimedia Appendix 4).

#### How do High-Risk Comorbidities Co-Occur Within and Across Subgroups of Readmitted Patients With HFx?

**Figure 2:**
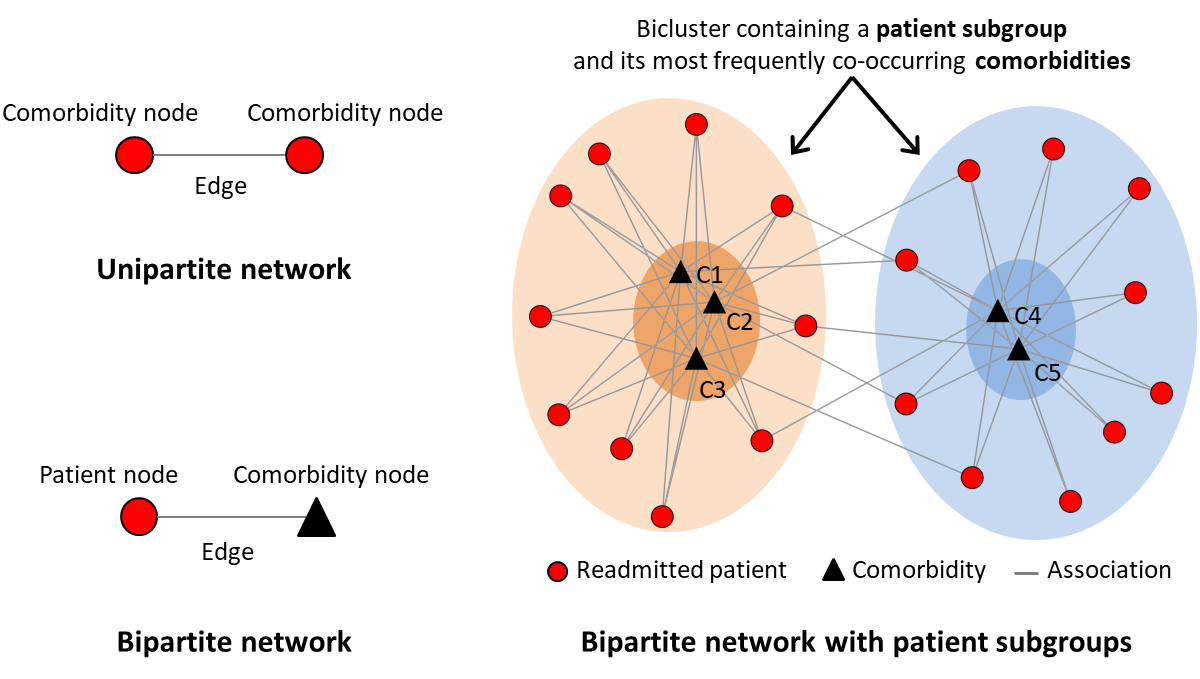
The distinction between a unipartite network, a bipartite network, and how the latter can be used to identify biclusters of patients and comorbidities.

To analyze how the above significant and replicated pairs co-occurred in readmitted patients with HFx, we used unsupervised bipartite networks. As shown in Figure 2, a network consists of nodes and edges; nodes represent one or more types of entities (eg, patients or comorbidities), and edges between the nodes represent a specific relationship between the entities. As shown in the upper left-hand part of Figure 2, a unipartite network has nodes that are of the same type (typically used to analyze co-occurrence of comorbidities [21]). In contrast, as shown in the lower left-hand part of Figure 2, a bipartite network has nodes that are of 2 types, and edges exist only between the 2 types, such as between patients (circles) and comorbidities (triangles). This quantitative and visual representation, which integrates patients and their comorbidities in a single representation, enables stakeholders to infer the mechanisms in each patient subgroup, a corner stone of precision medicine.

To analyze the data, we used the following steps: (1) represented the data as a bipartite network where nodes represented either patients or comorbidities, and the edges represented the presence or absence of a comorbidity; (2) identified patient subgroups and their most frequently co-occurring comorbidities using bicluster modularity [27,28] and tested its significance through comparisons with 1000 random permutations of the data; (3) used the Rand index (RI) [29] to measure the similarity of comorbidity co-occurrence between the training and replication data sets, and tested the RI significance; and (4) used the *ExplodeLayout* algorithm [30] to separate the biclusters, with the goal of reducing the visual overlap among them, thereby enhancing their comprehensibility.

#### What is the Relationship Between Comorbidity Risk, Comorbidity Co-Occurrence, and Patient Subgroups?

##### CoRisk Network Analysis: Integration of Risk, Co-Occurrence, and Patient Subgroups

The results from the supervised risk analysis and the unsupervised bipartite network analysis were integrated into a single network visualization. This was achieved by representing the high-risk and replicated pairs and their direction (identified in research question 1) using a directed unipartite network, where nodes represented the comorbidities and directed edges represented the direction of that risk. This unipartite network was superimposed onto the bipartite network of readmitted patients with HFx and comorbidities (described in research question 2) resulting in a *co-occurrence risk* (CoRisk) network. We define this CoRisk network visualization as the merging of 2 networks: (1) a bipartite network consisting of nodes representing patients and comorbidities, with edges representing their pairwise relationship and (2) a comorbidity risk network consisting of weighted directed edges between the comorbidities representing the risk and direction of significant and replicated comorbidity pairs. This integration of the supervised and unsupervised analytical results was designed to enable clinician stakeholders to interpret the relationship between high-risk pairs of comorbidities, their co-occurrence, and patient subgroups.

##### Clinical Interpretation of CoRisk Network

The CoRisk network was presented to a stakeholder team specializing in geriatrics and hospital re-admission, in addition to a biostatistician, who together examined the clinical meaningfulness of the risk, co-occurrence, and patient subgroups. The stakeholders were asked to visually analyze the CoRisk network and use their domain knowledge to (1) infer the underlying process that precipitated re-admission and (2) provide corroborative evidence from published literature to support their inferences.

## Results

In this section, we present the results of our analysis based on the 3 research questions:

### Which Combinations of Comorbidities Confer High Risk for Readmission in Patients With HFx?

As shown in Table 1, the *pairwise overall* *test* identified 24 pairs (all rows shown in the table) that were significant in the training data set. Furthermore, the *pairwise directionality test* identified 10 pairs that were significant in both directions, 13 that were significant only in one direction, and 1 that was not significant in either direction (for clarity, only significant results are shown for the pairwise directionality test in Table 1).

**Table 1:** The 24 comorbidity pairs that had significantly higher risk for readmission in the training data set, of which 11 replicated (serial number pairs 1-11) in the test data by being significant in the same direction.

Serial number	Comorbidity pair		Pairwise overall test		Pairwise directionality test				
	A	B	(A&B) vs (A + B + not A & not B)		(A&B) vs A			(A&B) vs B	
			OR^a^ (95% CI)	False discovery rate *P* valu	OR (95% CI)	False discovery rate *P* value	OR (95% CI)		False discovery rate *P* value
1	CHF^b^	COPD^c^	1.62 (1.40-1.89)	<.001	1.24 (1.05-1.47)	.019	1.36 (1.15-1.61)		.004
2	CHF	MCMCT^d^	1.81 (1.46-2.25)	<.001	1.38 (1.10-1.73)	.01	1.33 (1.05-1.69)		.03
3	CHF	Renal failure (I-V)	1.49 (1.31-1.70)	<.001	NS^e^	NS	1.34 (1.15-1.55)		.003
4	CHF	Stroke	1.99 (1.40-2.83)	.005	1.50 (1.05-2.14)	.04	NS		NS
5	Diabetes (without complications)	CHF	1.47 (1.25-1.73)	<.001	1.53 (1.28-1.83)	.000	NS		NS
6	Arrhythmias	Renal failure (I-V)	1.38 (1.20-1.60)	.001	NS	NS	1.20 (1.02-1.41)		.04
7	RF(I-V)	MCMCT	1.59 (1.28-1.96)	.001	1.37 (1.10-1.71)	.01	NS		NS
8	COPD	Renal failure (I-V)	1.46 (1.22-1.74)	.002	NS	NS	1.26 (1.04-1.52)		.03
9	Diabetes (without complications)	COPD	1.47 (1.21-1.77)	.004	1.49 (1.22-1.83)	.001	NS		NS
10	Stroke	Renal failure (I-V)	2.39 (1.55-3.69)	.004	NS	NS	2.04 (1.32-3.17)		.005
11	Vascular disease	MCMCT	2.03 (1.39-2.98)	.009	1.70 (1.13-2.53)	.02	NS		NS
12	CHF	Arrhythmias	1.46 (1.30-1.64)	<.001	NS	NS	1.25 (1.09-1.43)		.005
13	Arrhythmias	COPD	1.62 (1.38-1.89)	<.001	1.36 (1.15-1.62)	.002	1.34 (1.12-1.60)		.005
14	Arrhythmias	Stroke	2.23 (1.63-3.04)	<.001	1.85 (1.35-2.54)	.001	NS		NS
15	Stroke	COPD	3.18 (1.87-5.41)	.001	2.03 (1.15-3.60)	.02	2.56 (1.50-4.36)		.004
16	Arrhythmias	Hemiplegia/hemiparesis	2.18 (1.51-3.16)	.002	1.80 (1.24-2.62)	.006	2.12 (1.38-3.25)		.004
17	Angina	Arrhythmias	1.85 (1.38-2.49)	.003	1.92 (1.38-2.68)	.001	1.53 (1.13-2.07)		.02
18	CHF	Hemiplegia/hemiparesis	2.25 (1.49-3.40)	.005	1.70 (1.12-2.57)	.02	2.11 (1.33-3.34)		.005
19	Cardio-respiratory failure	CHF	1.65 (1.28-2.13)	.005	1.70 (1.25-2.31)	.003	NS		NS
20	Vascular disease	Renal failure (I-V)	1.63 (1.27-2.10)	.005	1.39 (1.04-1.85)	.04	1.40 (1.08-1.81)		.02
21	COPD	MCMCT	1.58 (1.24-2.03)	.01	NS	NS	NS		NS
22	Septicemia/shock	Renal failure (I-V)	2.51 (1.49-4.22)	.02	2.85 (1.47-5.52)	.006	2.14 (1.27-3.61)		.01
23	Intestinal obstruction	Arrhythmias	2.36 (1.45-3.84)	.02	2.17 (1.25-3.77)	.01	1.94 (1.18-3.17)		.02
24	Stroke	Hemiplegia/hemiparesis	1.78 (1.26-2.53)	.04	NS	NS	1.63 (1.08-2.46)		.03

^a^OR: odds ratio.

^b^CHF: congestive heart failure.

^c^COPD: chronic obstructive pulmonary disease.

^c^MCMCT: major complications of medical care and trauma.

^d^NS: not significant.

Next, we identified which of the above 24 pairs replicated by identifying comorbidity pairs that were identical in their significance and direction in the replication data set. As shown in Table 1, of the 24 pairs, 11 pairs (highlighted in blue and pink) replicated in the replication data set. Of these, 2 pairs (serial number pairs 1-2) were significant in both directions, and 9 pairs (serial number pairs 3-11) were significant only in one direction. The overlapping pairs resulted in 8 unique comorbidities: congestive heart failure (CHF), COPD, major complications of medical care and trauma (MCMCT), RF I-V, stroke, diabetes without complications (diabetes), arrhythmias, and vascular disease.

### How Do High-Risk Comorbidities Co-Occur Within and Across Subgroups of Readmitted Patients With HFx?

#### Visualization

**Figure 3:**
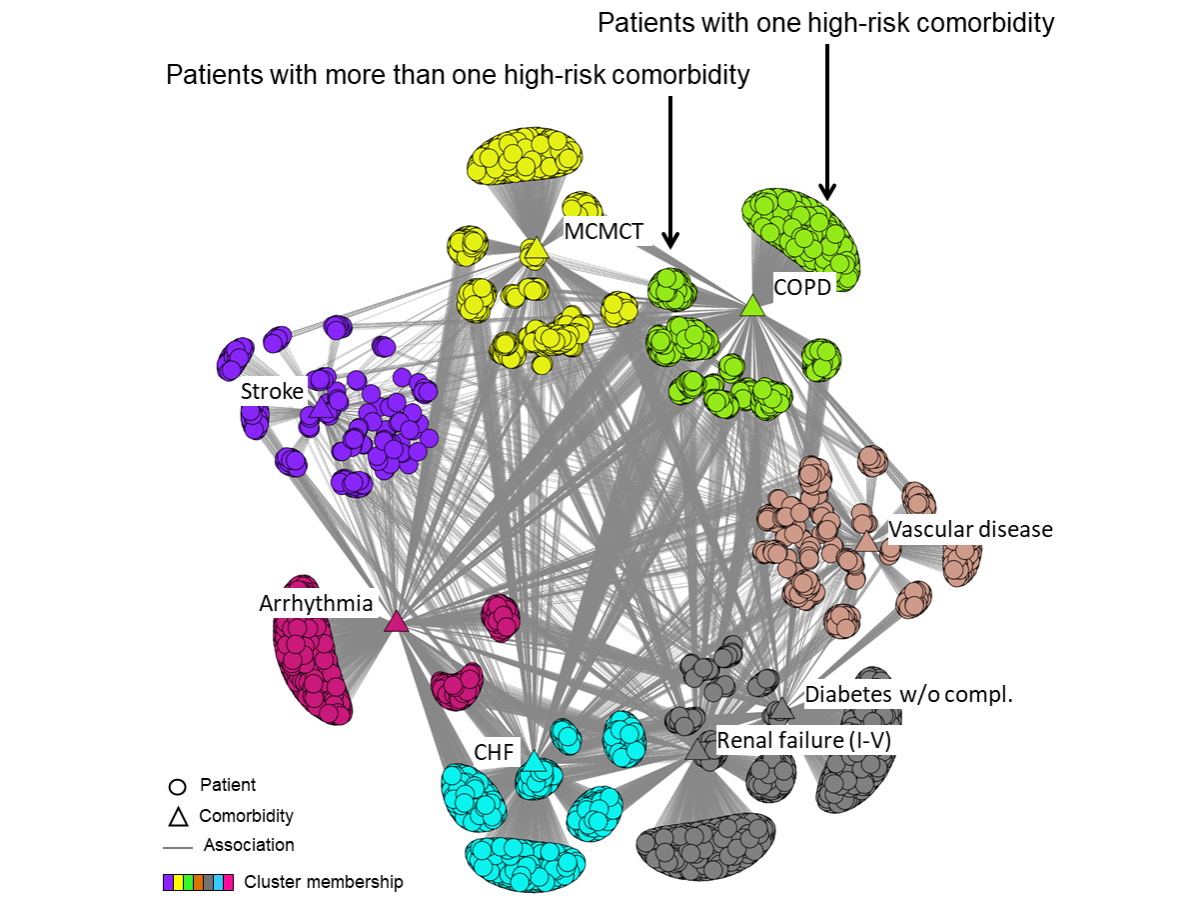
Bipartite network of significant and replicated comorbidities and re-admitted patients with HFx from the training data set.

To comprehend how high-risk comorbidities co-occurred across patients, we conducted a bipartite network analysis. The nodes consisted of the 8 significant and replicated comorbidities implicated in risk for readmission from the above combinatorial analysis, and all readmitted patients with HFx with at least one of those comorbidities (n=6150). As shown in Figure 3, the bipartite network analysis revealed 7 biclusters of patients and high-risk comorbidities.

#### Quantitative Verification and Layout Refinement

The network had a modularity of 0.440, which was significant (*P*<.001; Medicare=0.440; random mean 0.383 [0.002]) compared to 1000 random permutations of the network while preserving the network size (number of nodes) and network density (number of edges). The corresponding network generated from the replication data set also had 7 biclusters, a modularity of 0.444, which was also highly significant (*P*<.001; Medicare=0.444; random mean 0.379 [0.002]) compared to 1000 random permutations of the data while preserving network size and density.

#### Replication of Modularity and Comorbidity Co-Occurrence

The co-occurrence of comorbidities within and across clusters (as measured by the RI) between the training and replication data sets was significant (*P*=.02; Medicare=0.929; random mean 0.869 [0.027]), indicating a strong similar and significant co-occurrence pattern of comorbidities in the 2 networks. The training and test bipartite networks were therefore strongly biclustered (as measured by the similarly high biclustered modularity), highly significant (as measured by the permutation test), had a similar pattern of co-occurrence (as measured by the RI and its significance), and had the same number (7) of biclusters.

Although the above quantitative analysis revealed a significant and replicated overall clustered topology, a visual analysis of the network revealed 2 important patterns related to comorbidity co-occurrence and heterogeneity within patient subgroups:

##### Comorbidity Co-Occurrence

As shown in Figure 3, 6 comorbidities belonged to single-comorbidity biclusters, whereas 2 comorbidities co-occurred in the same cluster. This indicates that although many patients in one bicluster had comorbidities in another (as shown by the many edges between the clusters), the bicluster overlap in most cases was not strong enough to pull comorbidities into the same bicluster. One exception was the bicluster with RF and diabetes, where there were many patients with both, resulting in them being pulled together into the same bicluster.

##### Heterogeneity Within Patient Subgroups

As shown in Figure 3, each bicluster had a set of patients with only one comorbidity (in the outer side of the bicluster), and another set of patients with more than one comorbidity (in the inner side of the bicluster), revealing an additional level of heterogeneity within each bicluster. As shown in Table 2, the biclusters had different proportions of one or more comorbidities. For example, only 30% of patients in the arrhythmia bicluster had more than one high-risk comorbidity compared with 78% of patients in the vascular disease cluster. This bicluster-specific heterogeneity, as measured by the ratio of patients with one to many comorbidities, was significantly different across the 7 biclusters (*X*^2^_6_=868.6; N=6150; *P*<.001).

The bipartite network analysis therefore not only revealed how the comorbidities co-occurred across patient subgroups but also the patient heterogeneity at the network-wide level and at the bicluster-specific level, revealing the real-world variations in the comorbidity profiles of patients with HFx.

**Table 2:** The number of patients with one or more comorbidities across the 7 biclusters (patients with one comorbidity in the RF and diabetes bicluster had either RF or diabetes).

Number of comorbidities	CHF^a^, n (%)	Arrhythmia, n (%)	Stroke, n (%)	MCMCT^b^, n (%)	COPD^c^, n (%)	Vascular disease, n (%)	Renal failure and diabetes, n (%)	Total, n (%)
Comorbidities=1	536 (50)	545 (69.8)	37 (13.7)	337 (39.4)	510 (41.3)	114 (21.7)	1062 (75.32)	3141 (51.07)
Comorbidities>1	536 (50)	236 (30.2)	233 (86.3)	518 (60.6)	726 (58.7)	412 (78.3)	348 (24.7)	3009 (48.93)
Total	1072 (100)	781 (100)	270 (100)	855 (100)	1236 (100)	526 (100)	1410 (100)	6150 (100)

^a^CHF: congestive heart failure.

^b^MCMCT: major complications of medical care and trauma.

^c^COPD: chronic obstructive pulmonary disease.

### What is the Relationship Between Comorbidity Risk, Comorbidity Co-Occurrence, and Patient Subgroups?

**Figure 4:**
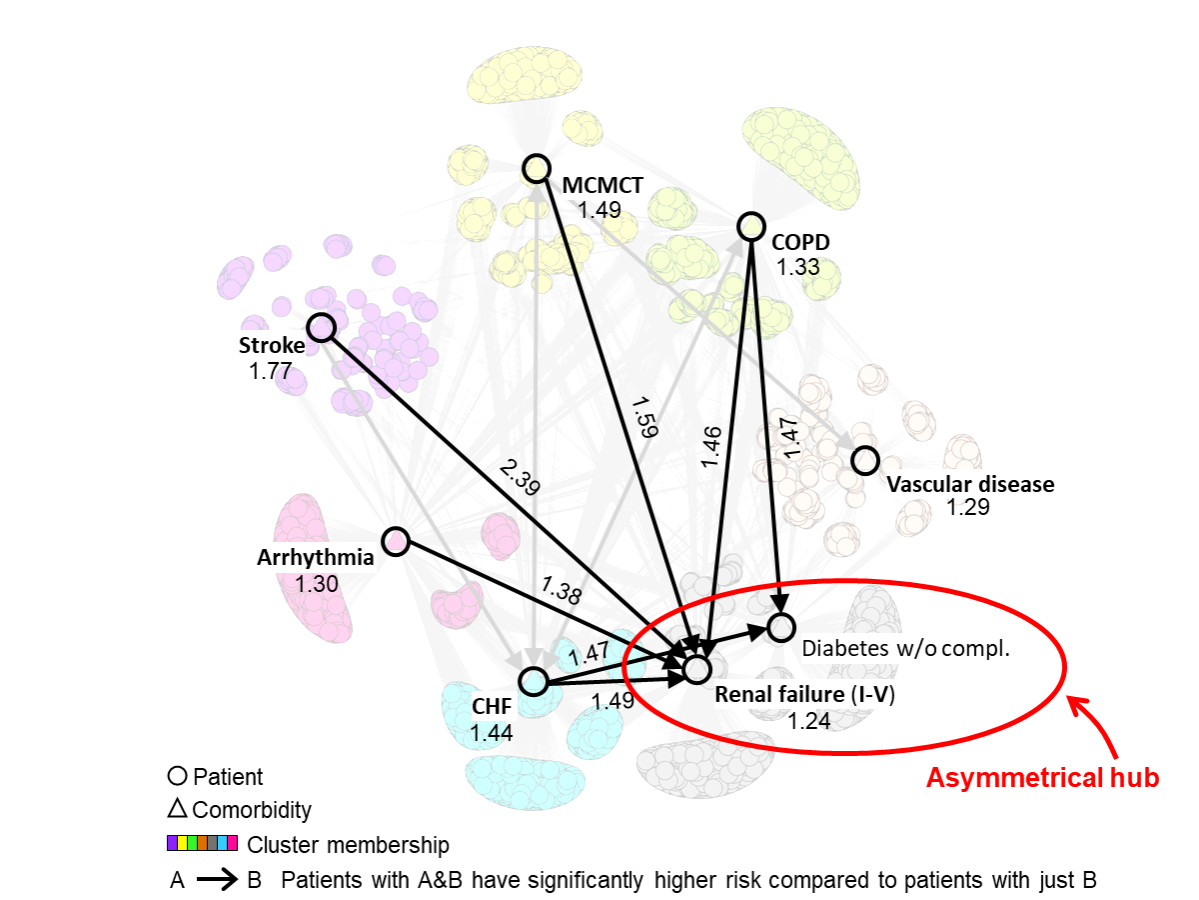
CoRisk network showing the integrated results from the supervised combinatorial analysis and the unsupervised bipartite network analysis. The numbers on the nodes refer to the odds ratios of comorbidities that were significantly associated to 30-day readmissions, the numbers on the edges refer to the ORs of pairs that were significant based on the pairwise overall test, and the direction of the edges represent the pairs that were significant and replicated in the same direction based on the pairwise directionality test. CHF: congestive heart failure; COPD: chronic obstructive pulmonary disease; w/o compl: without complications; MCMCT: major complications of medical care and trauma.

As shown in Figure 4, the CoRisk network revealed how the high-risk pairs were (1) related to each other, (2) their directionality, and (3) how they were related to the patient subgroups. This integrated network enabled stakeholders to identify 2 sets of comorbidities. The first set (diabetes and RF) consisted of comorbidities that can have multi-organ consequences and is therefore referred to as systemic diseases. In contrast, the second set (CHF, arrhythmia, stroke, MCMCT, COPD, and vascular disease) consisted of comorbidities that had mainly organ-specific consequences. For example, while cardiac arrhythmia could potentially have systemic consequences, this comorbidity is specific to the electrophysiological properties of the heart.

As the clinician stakeholders were most interested in the interrelationship of risk between multi-organ and organ-specific comorbidities, we bolded all the edges that started from an organ-specific comorbidity (CHF, arrhythmia, stroke, MCMCT, and COPD) and ended at a multi-organ comorbidity (RF or diabetes). As shown in Figure 4, all the remaining edges pointed toward RF and diabetes, forming an *asymmetrical hub* (more edges pointing in than those pointing out). This meant that the implicated pairs connecting the nodes had significantly higher risk compared with RF and diabetes alone, but not significantly higher risk compared with the other members of the pair. For example, the directed edge starting from COPD and pointing to diabetes indicated that patients with COPD and diabetes have a significantly higher risk compared with diabetes alone, but not a significantly higher risk compared with COPD alone.

The asymmetrical risk hubs of RF and diabetes suggested that because they have multi-organ consequences, their outcomes are largely chronic and therefore require considerable severity on their own before they become the sole risk factors for readmission (note that the HCC definition of RF has a wide range from Stage I to V, possibly resulting in several patients with RF being in the early stages). However, when they co-occur with an organ-specific disease such as CHF, arrhythmia, stroke, MCMCT, or COPD, it can exacerbate those pre-existing conditions leading to a significantly higher risk of readmission. This pattern of asymmetrical risks resulted in the following hypothesis for a 2-tiered comorbidity exacerbation risk model with significantly higher risk at each subsequent tier:

1. Tier 1 risk (only multi-organ comorbidities): RF, diabetes. This tier consists of patients in the RF-diabetes cluster.
2. Tier 2 risk (multi-organ plus organ-specific comorbidities): RF plus CHF or arrhythmia or stroke or MCMCT or COPD (patients in the inner part of the biclusters) in addition to patients with CHF, arrhythmia, stroke, MCMCT, or COPD (patients in the outer part of the respective biclusters).

Combining the above risk model with their own domain experience, the physician and the occupational therapist on the stakeholder team inferred hypotheses for the processes precipitating readmission in patients with HFx and provided corroborative evidence from the literature to support their inferences. They noted that when a patient is discharged from a hospital after an HFx surgery, the standard-of-care in generating discharge notes and order sets is focused on wound healing, postoperative delirium, mobilization, rehabilitation, and nutritional needs [31,32]. However, despite these guidelines, older adult patients, particularly those in skilled nursing facilities, regularly suffer from dehydration and malnutrition [33-37]. These conditions can worsen compromised renal function [38] as well as glycemic control in diabetics, ultimately triggering the deterioration of existing organ-specific comorbidities such as CHF and COPD [39-41]. Unfortunately, by the time symptoms of exacerbation in comorbidities are detected, the patient’s health may have considerably declined, requiring urgent care, triggering an unplanned hospital readmission.

The nurse practitioner on the stakeholder team further stated that a contributing factor to the above cascade of events could be the lack of multidisciplinary care when patients with HFx are discharged after surgery. As stated in a recent review [42], HFx management “requires physicians to anticipate problems that may arise during recovery, whether the complications are from hip fracture and immobility, exacerbations of chronic diseases, or problems with social and psychological support...it takes a team of dedicated professionals working together seamlessly to deliver care appropriate for patient goals, and to maximize recovery.” In fact, although an increase in the number of registered nurses and multidisciplinary care teams has been associated with reduced 30-day readmission rates and improved health outcomes [43-45], such post–acute care is not yet widespread. The results of the analysis, combined with domain experience and corroborating evidence, enabled the clinician stakeholders to infer that the generation of discharge notes and order sets *before* discharge and the level of multidisciplinary care *after* discharge could be prime targets for reducing the risk of hospital readmission in specific subgroups of patients with HFx.

## Discussion

### Implications for Designing Targeted Interventions and Predictive Models

Our approach to integrate the results from supervised and unsupervised approaches into the CoRisk network helped to reveal (1) the overlap among the high-risk pairs, resulting in the stakeholders identifying the asymmetrical hub, and (2) the relationship of high-risk pairs to the network-wide and bicluster-specific patient heterogeneity. These results enabled the clinician stakeholders to infer hypotheses about the processes that precipitate readmission through a comorbidity exacerbation risk model. These results have the following implications for the design of interventions and predictive modeling.

#### Design of Postoperative Interventions

When a patient with HFx is discharged, the discharge notes and order sets could state which of the seven high-risk pre-existing comorbidities exist in the patient, with the respective recommendations for recognizing the early signs of the worsening of those comorbidities. For example, patients with RF should be monitored by rehabilitation or home health providers for urine output or weight gain, and those with diabetes should have more than usual monitoring of blood glucose during the convalescent period. However, patients with *both* RF and CHF should have their volume status more closely monitored, as they are more likely to develop an acute CHF exacerbation than patients with CHF alone. This is because patients with RF have a reduced ability to regulate the volume status and small fluctuations can precipitate acute CHF exacerbation, resulting in cardiorenal syndrome [46].

Furthermore, rehabilitation providers (physical therapists, physicians, registered nurses, and social workers) should be specifically trained to recognize and report changes in physical status, such as reduced oral intake, which might be an early warning of impending exacerbation of the specific comorbidities identified in the analysis. Finally, given the scarcity of rehab resources, clinicians could use the 2-tiered significant risk profile discussed above in triaging care, such as conducting more frequent evaluations of patients with HFx with COPD and RF compared with those with only RF. Future clinical trials could test whether improved discharge notes and order sets, in addition to early identification and treatment of worsening comorbidities through multidisciplinary team monitoring, can help to reduce the risk of readmission in patients with HFx.

#### Design of Preoperative Interventions

The results also suggest that combinations of high-risk comorbidities could be used to fine-tune the current criteria to select patients who should undergo HFx surgery. For example, certain comorbidity combinations could simply reflect the overall poor performance status of a patient, for whom postoperative interventions, no matter how robust, might end up being largely ineffective in preventing readmission. Future models could identify which subsets of patients have such *unmodifiable* readmission risks that outweigh the benefits of surgery and therefore could be better served with more conservative approaches.

#### Design of Predictive Models

The bipartite network analysis of patients and comorbidities showed significant and replicated heterogeneity among the readmitted patients based on their comorbidity profiles. However, current logistic regression models designed to predict readmission do not consider such heterogeneity in readmitted patients. For example, the regression model developed for CMS to predict readmission in arthroplasty or hip replacement patients [14] uses a single model to predict readmission for all patients. Although this model was an important advancement in predicting readmission in this population, it assumes that all patients can be modeled using a uniform set of coefficients for the same variables, an assumption that could conceal heterogeneity in readmitted patients and affect the accuracy of prediction in patient subgroups.

As stated by the biostatistician on the stakeholder team, a common approach to address such heterogeneity is to develop stratified regression models [47,48], one for each stratum of the population. The mathematical intuition underlying stratified regression models is that regression models can achieve a better fit to subsets of the data that are homogenous compared with a single regression model that is fitted to all of the data. For example, recognizing that races have different risks for developing type 2 diabetes, a recent study demonstrated that race-stratified regression models resulted in improved prediction accuracy for a racial subgroup [47]. However, such patient stratifications are typically selected based on an *a priori* understanding of the domain, which might miss important patterns in the data.

In contrast to the above approach of selecting patient subgroups, we believe our approach can enable the automatic identification of patient stratification that is data-driven and furthermore tested for significance and replicability, as we have demonstrated. Such information could then be used to develop stratified regression models to test whether they reveal heterogeneity in prediction accuracy for one or more patient subgroups. For example, stratified regression models could be developed and tested for each of the 7 clusters shown in Figure 3. Furthermore, given that each of the clusters had an outer subgroup (with only one high-risk comorbidity) and an inner subgroup (with more than one high-risk comorbidity), future regression models could also be targeted at each of these subgroups within biclusters, depending on their prevalence. Finally, each of the above regression models could test for interactions among the 11 high-risk comorbidity pairs shown in Table 1.

Improvements achieved through stratified regression models are dependent on a host of factors, including the degree of homogeneity in patient subgroups, the adequacy of sample size within those subgroups, and the tradeoff between prediction accuracy and model complexity. Future research should, therefore, determine whether stratified regression models based on automatically identified patient subgroups can produce more robust predictive models for hospital readmission.

### Strengths and Limitations

The strength of this study is that we integrated the results from well-known methods with novel approaches, which together enabled a deeper understanding of the associations between risk, co-occurrence, and subgroups. This in turn led to insights related to targeted interventions (a critical goal of precision medicine), in addition to the design of predictive models. Furthermore, the analytical results were replicated in another year, demonstrating its generalizability. Critical to this process was the team-centered informatics approach [22] we pursued at each step of the project, which used intuitive visual analytical representations to span the disciplinary boundaries of clinicians and methodology stakeholders, enabling them to comprehend and address the complexity in a large data set.

A limitation of this study is that we tested the method on just one index condition, and our ongoing research [49] is testing the approach on other index conditions. Furthermore, the interpretability of the clusters could be enhanced by constructing additional figures wherein the patient nodes are colored based on covariates important to hospital readmission (eg, age, gender, race, length of hospital stay, and reason for readmission), in addition to determining which of them are significantly higher and lower across the clusters. Finally, the prevalence and severity of comorbidities may vary in patients receiving care in clinics, acute care hospitals, skilled nursing facilities, and nursing home settings. Therefore, future research should analyze whether the results vary across different care settings.

Fully cognizant that few data sets are without limitations, we consciously chose to analyze Medicare data because of its scale (enabling us to have adequate numbers of patients when analyzing patient heterogeneity), availability of data over multiple years (enabling us to test external replicability), and generalizability (enabling us to analyze data from patients and hospitals across the United States). However, given that Medicare data are collected mainly for administrative purposes, it has well-known limitations, including the lack of test results, which could enable a finer understanding of the severity of comorbidities. Furthermore, comorbidities associated with mental health are known to be undercoded in the Medicare database, which could bias our results. Therefore, when clinical data across hospitals become available in future (eg, through the PCORnet [50] funded by the Patient-Centered Outcomes Research Institute [PCORI] and through the Accrual to Clinical Trials network [51] funded by the National Center for Advancing Translational Sciences [NCATS]), we intend to repeat our analysis using clinical data, but we fully realize that such data might have limitations that are yet unknown.

Methodologically, our approach of integrating supervised and unsupervised visual analytical approaches is just one of the many possible ways such integration can be achieved [52]. In our future research, we plan to explore other integration strategies with a specific focus on enabling clinician stakeholders to go beyond the analyses of prevalence and risk, enabling inferences for the underlying processes precipitating readmission. Such improvements in data and methods should enable discharge planners and providers in rehabilitation facilities to more accurately predict which patients will be readmitted and to select targeted interventions to reduce the risk of readmission and, consequently, the concomitant burden on patients and caregivers.

## Appendix

Multimedia Appendix 1 Number of patients based on the selection criteria.

Multimedia Appendix 2 The 70 HCC codes that represent the range of comorbidities typically encountered in the elderly.

Multimedia Appendix 3 Cumulative frequency of comorbidities in HFx patients. The analysis revealed that 32 comorbidities together accounted for <1% of the patients, and were removed from the data, with the remaining 37 comorbidities used for the subsequent analyses.

Multimedia Appendix 4 R (supported by the R Foundation for Statistical Computing) packages and functions used for the analyses.

## Abbreviations

CHF: congestive heart failure
CMS: Centers for Medicare & Medicaid Services
COPD: chronic obstructive pulmonary disease
CoRisk: co-occurrence risk
FDR: false discovery rate
HCCs: hierarchical condition categories
HFx: hip fracture
HRRP: Hospital Readmissions Reduction Program
MCMCT: major complications of medical care and trauma
MS-DRG: Medicare Severity-Diagnosis Related Group
NCATS: National Center for Advancing Translational Sciences
PCORI: Patient-Centered Outcomes Research Institute
RF: renal failure
RI: Rand index

## References

[1] Hip Fractures Among Older Adults. Centers for Disease Control and Prevention.

[2] French DD, Bass E, Bradham DD, Campbell RR, Rubenstein LZ (2008). Rehospitalization after hip fracture: predictors and prognosis from a national veterans study. J Am Geriatr Soc.

[3] (2013). The Revolving Door: A Report on US Hospital Readmissions. The Robert Wood Johnson Foundation.

[4] Jencks SF, Williams MV, Coleman EA (2009). Rehospitalizations among patients in the medicare fee-for-service program. N Engl J Med.

[5] Ashton CM, Del Junco DJ, Souchek J, Wray NP, Mansyur CL (1997). The association between the quality of inpatient care and early readmission: a meta-analysis of the evidence. Med Care.

[6] Fonarow GC, Konstam MA, Yancy CW (2017). The hospital readmission reduction program is associated with fewer readmissions, more deaths: time to reconsider. J Am Coll Cardiol.

[7] Wasfy JH, Zigler CM, Choirat C, Wang Y, Dominici F, Yeh RW (2017). Readmission rates after passage of the hospital readmissions reduction program: a pre-post analysis. Ann Intern Med.

[8] Wadhera RK, Maddox KE, Wasfy JH, Haneuse S, Shen C, Yeh RW (2018). Association of the hospital readmissions reduction program with mortality among medicare beneficiaries hospitalized for heart failure, acute myocardial infarction, and pneumonia. J Am Med Assoc.

[9] Ody C, Msall L, Dafny LS, Grabowski DC, Cutler DM (2019). Decreases in readmissions credited to medicare's program to reduce hospital readmissions have been overstated. Health Aff (Millwood).

[10] Boockvar KS, Halm EA, Litke A, Silberzweig SB, McLaughlin M, Penrod JD, Magaziner J, Koval K, Strauss E, Siu AL (2003). Hospital readmissions after hospital discharge for hip fracture: surgical and nonsurgical causes and effect on outcomes. J Am Geriatr Soc.

[11] Pollock FH, Bethea A, Samanta D, Modak A, Maurer JP, Chumbe JT (2015). Readmission within 30 days of discharge after hip fracture care. Orthopedics.

[12] Härstedt M, Rogmark C, Sutton R, Melander O, Fedorowski A (2015). Impact of comorbidity on 6-month hospital readmission and mortality after hip fracture surgery. Injury.

[13] Cram P, Lu X, Kaboli PJ, Vaughan-Sarrazin MS, Cai X, Wolf BR, Li Y (2011). Clinical characteristics and outcomes of medicare patients undergoing total hip arthroplasty, 1991-2008. J Am Med Assoc.

[14] (2015). 2015 Procedure-specific Readmission Measures Updates and Specifications Report: Elective Primary Total Hip Arthroplasty and/or Total Knee Arthroplasty, and Isolated Coronary Artery Bypass Graft Surgery. Centers for Medicare and Medicaid Services.

[15] Aryal S, Diaz-Guzman E, Mannino D (2013). Prevalence of COPD and comorbidity. Eur Respir Monogr.

[16] Baty F, Putora PM, Isenring B, Blum T, Brutsche M (2013). Comorbidities and burden of COPD: a population based case-control study. PLoS One.

[17] Moni MA, Liò P (2014). Network-based analysis of comorbidities risk during an infection: SARS and HIV case studies. BMC Bioinformatics.

[18] Cramer AO, Waldorp LJ, van der Maas HL, Borsboom D (2010). Comorbidity: a network perspective. Behav Brain Sci.

[19] Lochner KA, Cox CS (2013). Prevalence of multiple chronic conditions among medicare beneficiaries, United States, 2010. Prev Chronic Dis.

[20] Islam MM, Valderas JM, Yen L, Dawda P, Jowsey T, McRae IS (2014). Multimorbidity and comorbidity of chronic diseases among the senior Australians: prevalence and patterns. PLoS One.

[21] Folino F, Pizzuti C, Ventura M (2010). A Comorbidity Network Approach to Predict Disease Risk. Proceedings of the International Conference on Information Technology in Bio- and Medical Informatics.

[22] Bhavnani SK, Visweswaran S, Divekar R, Brasier AR (2018). Towards team-centered informatics: accelerating innovation in multidisciplinary scientific teams through visual analytics. J Appl Behav Sci.

[23] (2013). Report to the Congress: Medicare and the Health Care Delivery System. Chapter 3. Approaches to Bundle Payment for Post-Acute Care Washington. Medicare Payment Advisory Commission (MedPAC).

[24] Pope G, Kautter J, Ingber M, Freeman S, Sekar R, Newhart C (2011). Evaluation of the CMS-HCC Risk Adjustment Model. Centers for Medicare & Medicaid Services' Office of Research, Development, and Information.

[25] St Sauver JL, Boyd CM, Grossardt BR, Bobo WV, Finney Rutten LJ, Roger VL, Ebbert JO, Therneau TM, Yawn BP, Rocca WA (2015). Risk of developing multimorbidity across all ages in an historical cohort study: differences by sex and ethnicity. BMJ Open.

[26] Benjamini Y, Hochberg Y (2018). Controlling the false discovery rate: a practical and powerful approach to multiple testing. J R Stat Soc B.

[27] Treviño S, Nyberg A, del Genio CI, Bassler KE (2015). Fast and accurate determination of modularity and its effect size. J Stat Mech.

[28] Chauhan R, Ravi J, Datta P, Chen T, Schnappinger D, Bassler KE, Balázsi G, Gennaro ML (2016). Reconstruction and topological characterization of the sigma factor regulatory network of Mycobacterium tuberculosis. Nat Commun.

[29] Rand WM (1971). Objective criteria for the evaluation of clustering methods. J Am Stat Assoc.

[30] Bhavnani SK, Chen T, Ayyaswamy A, Visweswaran S, Bellala G, Rohit D, Kevin E (2017). Enabling comprehension of patient subgroups and characteristics in large bipartite networks: implications for precision medicine. AMIA Jt Summits Transl Sci Proc.

[31] Mak JC, Cameron ID, March LM, National Health and Medical Research Council (2010). Evidence-based guidelines for the management of hip fractures in older persons: an update. Med J Aust.

[32] Rao S, Cherukuri M (2006). Management of hip fracture: the family physician's role. Am Fam Physician.

[33] Mentes JC, Chang BL, Morris J (2006). Keeping nursing home residents hydrated. West J Nurs Res.

[34] Bennett JA (2000). Dehydration: hazards and benefits. Geriatr Nurs.

[35] Lelovics Z (2009). [Nutritional status and nutritional rehabilitation of elderly people living in long-term care institutions]. Orv Hetil.

[36] Guigoz Y, Lauque S, Vellas BJ (2002). Identifying the elderly at risk for malnutrition. The mini nutritional assessment. Clin Geriatr Med.

[37] Shipman D, Hooten J (2007). Are nursing homes adequately staffed? The silent epidemic of malnutrition and dehydration in nursing home residents. Until mandatory staffing standards are created and enforced, residents are at risk. J Gerontol Nurs.

[38] Teixeira A, Trinquart L, Raphael M, Bastianic T, Chatellier G, Holstein J (2009). Outcomes in older patients after surgical treatment for hip fracture: a new approach to characterise the link between readmissions and the surgical stay. Age Ageing.

[39] Ronco C, Haapio M, House AA, Anavekar N, Bellomo R (2008). Cardiorenal syndrome. J Am Coll Cardiol.

[40] Fabbian F, de Giorgi A, Manfredini F, Lamberti N, Forcellini S, Storari A, Gallerani M, Caramori G, Manfredini R (2016). Impact of renal dysfunction on in-hospital mortality of patients with severe chronic obstructive pulmonary disease: a single-center Italian study. Int Urol Nephrol.

[41] Heyes GJ, Tucker A, Marley D, Foster A (2015). Predictors for readmission up to 1 year following hip fracture. Arch Trauma Res.

[42] Hung WW, Egol KA, Zuckerman JD, Siu AL (2012). Hip fracture management: tailoring care for the older patient. J Am Med Assoc.

[43] Forsythe L, Murray C, Shah B (2018). Effectiveness of interprofessional care teams on reducing hospital readmissions in patients with heart failure: a systematic review. MedSurg Nurs.

[44] Lasater KB, Mchugh MD (2016). Nurse staffing and the work environment linked to readmissions among older adults following elective total hip and knee replacement. Int J Qual Health Care.

[45] Ouslander JG, Berenson RA (2011). Reducing unnecessary hospitalizations of nursing home residents. N Engl J Med.

[46] Silverberg D, Wexler D, Blum M, Schwartz D, Iaina A (2004). The association between congestive heart failure and chronic renal disease. Curr Opin Nephrol Hypertens.

[47] Lacy ME, Wellenius GA, Carnethon MR, Loucks EB, Carson AP, Luo X, Kiefe CI, Gjelsvik A, Gunderson EP, Eaton CB, Wu W (2016). Racial differences in the performance of existing risk prediction models for incident type 2 diabetes: the cardia study. Diabetes Care.

[48] Tan A, Kuo Y, Goodwin JS (2013). Predicting life expectancy for community-dwelling older adults from medicare claims data. Am J Epidemiol.

[49] Bhavnani S, Lin Y, Chennuri L, Bores J, Chen C, Kuo Y (2018). Identification, Replication, Visualization, and Interpretation of Patient Subgroups: Implications for Precision Medicine, and Predictive Modeling. AMIA Joint Summits on Translational Science Proceedings AMIA Summit on Translational Science.

[50] Fleurence RL, Curtis LH, Califf RM, Platt R, Selby JV, Brown JS (2014). Launching PCORnet, a national patient-centered clinical research network. J Am Med Inform Assoc.

[51] Amin W, Borromeo C, Saul M, Becich M, Visweswaran S (2015). Informatics Synergies Between PaTH and ACT Networks. AMIA Annual Symposium proceedings / AMIA Symposium AMIA Symposium.

[52] Shneiderman B (2002). Inventing discovery tools: combining information visualization with data mining. Inf Vis.

