# Supplementary material for: How High-Risk Comorbidities Co-Occur in Readmitted Patients With Hip Fracture: Big Data Visual Analytical Approach
Source: JMIR Med Inform. 2020 Oct 26;8(10):e13567. doi: 10.2196/13567 (PMC7652691; doi:10.2196/13567)
Supplement: Multimedia Appendix 1 [file medinform_v8i10e13567_app1.docx]

| Selection Criteria | | | | | | N |
| --- | --- | --- | --- | --- | --- | --- |
| Eligible Sample: Index Acute Hospitalization for MS-DRG (2009-2011) | | | | | | 3,030,042 |
| Discharge to: IRF/SNF/HHA | | | | | | 2,248,598 |
| Cases d/c on or before September 2011 | | | | | | 2,061,459 |
| Link with Beneficiary Summary file | | | | | | 2,061,427 |
| Medicare Part A coverage | | | | | | 1,875,452 |
| No HMO coverage | | | | | | 1,452,340 |
| Age 66-100 | | | | | | 1,321,334 |
| Survive 90-days post Index acute discharge | | | | | | 1,314,366 |
| Not transfer from SNF and admission is either Elective, Urgent or Emergency | | | | | | 1,293,442 |
| Index admission for DRG code in three years | | | | | | 1,253,603 |
| DRG codes: 480, 481, and 482 | | | | | | 245,346 |
| Unique ID for three years | | | | | | 241,148 |
| Delete Hospital Acquired Complications | | | | | | 202,417 |
| Remove joint replacements | | | | | | 202,415 |
| Remove cases admitted to long term acute care hospitals within 30 days | | | | | | 202,182 |
| Split by Discharge Years | | | | | | |
|  | 2009  (76,829) | | 2010  (72,786) | | 2011  (52,567) | |
|  | Cases | Control | Cases | Control |  | |
| 30-day unplanned readmissions, excluding controls with admissions to long-term acute care hospitals in 90-days | 8,699 | 55,830 | 9,104 | 53,663 |  |  |
| 1:1 Matching on age, gender, and race/ethnicity (White, Black, Hispanic and Other) | 8,485 | 8,443 | 8,134 | 8,111 |  |  |
| Total selected | 8,443 | 8,443 | 8,111 | 8,111 |  | |

S1 Table. Number of patients based on selection criteria
