# Supplementary material for: How High-Risk Comorbidities Co-Occur in Readmitted Patients With Hip Fracture: Big Data Visual Analytical Approach
Source: JMIR Med Inform. 2020 Oct 26;8(10):e13567. doi: 10.2196/13567 (PMC7652691; doi:10.2196/13567)
Supplement: Multimedia Appendix 2 [file medinform_v8i10e13567_app2.docx]

| # | **Code** | **Description** |
| --- | --- | --- |
| 1 | HCC1 | HIV/AIDS |
| 2 | HCC2 | Septicemia/shock |
| 3 | HCC5 | Opportunistic infections |
| 4 | HCC7 | Metastatic cancer and acute leukemia |
| 5 | HCC8 | Lung, upper digestive tract, and other severe cancers |
| 6 | HCC9 | Lymphatic, head and neck, brain, and other major cancers |
| 7 | HCC10 | Breast, prostate, colorectal and other cancers and tumors |
| 8 | HCC15 | Diabetes with renal or peripheral circulatory manifestation. This HCC includes Type I or Type II diabetes mellitus. HCC15, HCC16, HCC17 and HCC18 are constrained to be equal. |
| 9 | HCC16 | Diabetes with neurologic or other specified manifestation. This HCC includes Type I or Type II diabetes mellitus. HCC15, HCC16, HCC17 and HCC18 are constrained to be equal. |
| 10 | HCC17 | Diabetes with acute complications. This HCC includes Type I or Type II diabetes mellitus. HCC15, HCC16, HCC17 and HCC18 are constrained to be equal. |
| 11 | HCC18 | Diabetes with ophthalmologic or unspecified manifestation. This HCC includes Type I or Type II diabetes mellitus. HCC15, HCC16, HCC17 and HCC18 are constrained to be equal. |
| 12 | HCC19 | Diabetes without complication. This HCC includes Type I or Type II diabetes mellitus. |
| 13 | HCC21 | Protein-calorie malnutrition |
| 14 | HCC25 | End-stage liver disease |
| 15 | HCC26 | Cirrhosis of liver |
| 16 | HCC27 | Chronic hepatitis |
| 17 | HCC31 | Intestinal obstruction/perforation |
| 18 | HCC32 | Pancreatic disease |
| 19 | HCC33 | Inflammatory bowel disease |
| 20 | HCC37 | Bone/joint/muscle infections/necrosis |
| 21 | HCC38 | Rheumatoid arthritis and inflammatory connective tissue disease |
| 22 | HCC44 | Severe hematological disorders |
| 23 | HCC45 | Disorders of immunity |
| 24 | HCC51 | Drug/alcohol psychosis. This HCC is excluded from institutional model because the estimated coefficient is less than 0 or the *t*-statistic is less than 1.0. |
| 25 | HCC52 | Drug/alcohol dependence. This HCC is excluded from institutional model because the estimated coefficient is less than 0 or the *t*-statistic is less than 1.0. |
| 26 | HCC54 | Schizophrenia |
| 27 | HCC55 | Major depressive, bipolar, and paranoid disorders |
| 28 | HCC67 | Quadriplegia, other extensive paralysis |
| 29 | HCC68 | Paraplegia |
| 30 | HCC69 | Spinal cord disorders/injuries |
| 31 | HCC70 | Muscular dystrophy. This HCC is excluded from institutional model because the estimated coefficient is less than 0 or the *t*-statistic is less than 1.0. |
| 32 | HCC71 | Polyneuropathy |
| 33 | HCC72 | Multiple sclerosis |
| 34 | HCC73 | Parkinson's and Huntington's diseases |
| 35 | HCC74 | Seizure disorders and convulsions |
| 36 | HCC75 | Coma, brain compression/anoxic damage |
| 37 | HCC77 | Respirator dependence/tracheostomy status |
| 38 | HCC78 | Respiratory arrest |
| 39 | HCC79 | Cardio-respiratory failure and shock |
| 40 | HCC80 | Congestive Heart Failure |
| 41 | HCC81 | Acute myocardial infarction |
| 42 | HCC82 | Unstable angina and other acute ischemic heart disease |
| 43 | HCC83 | Angina pectoris/old myocardial infarction |
| 44 | HCC92 | Specified heart arrhythmias |
| 45 | HCC95 | Cerebral hemorrhage |
| 46 | HCC96 | Ischemic or unspecified stroke |
| 47 | HCC100 | Hemiplegia/hemiparesis |
| 48 | HCC101 | Cerebral palsy and other paralytic syndromes. This HCC is excluded from institutional model because the estimated coefficient is less than 0 or the *t*-statistic is less than 1.0. |
| 49 | HCC104 | Vascular disease with complications |
| 50 | HCC105 | Vascular disease |
| 51 | HCC107 | Cystic fibrosis |
| 52 | HCC108 | Chronic Obstructive Pulmonary Disease |
| 53 | HCC111 | Aspiration and specified bacterial pneumonias |
| 54 | HCC112 | Pneumococcal pneumonia, emphysema, lung abscess |
| 55 | HCC119 | Proliferative diabetic retinopathy and vitreous hemorrhage |
| 56 | HCC130 | Dialysis status |
| 57 | HCC131 | Renal Failure |
| 58 | HCC132 | Nephritis |
| 59 | HCC148 | Decubitus ulcer of skin |
| 60 | HCC149 | Chronic ulcer of skin, except decubitus |
| 61 | HCC150 | Extensive third-degree burns. This HCC is excluded from institutional model because the estimated coefficient is less than 0 or the *t*-statistic is less than 1.0. |
| 62 | HCC154 | Severe head injury |
| 63 | HCC155 | Major head injury. This HCC is excluded from institutional model because the estimated coefficient is less than 0 or the *t*-statistic is less than 1.0. |
| 64 | HCC157 | Vertebral fractures without spinal cord injury |
| 65 | HCC158 | Hip fracture/dislocation. This HCC is excluded from institutional model because the estimated coefficient is less than 0 or the *t*-statistic is less than 1.0. |
| 66 | HCC161 | Traumatic amputation |
| 67 | HCC164 | Major Complications of Medical Care and Trauma |
| 68 | HCC174 | Major organ transplant status |
| 69 | HCC176 | Artificial openings for feeding or elimination |
| 70 | HCC177 | Amputation status, lower limb / amputation complications |
