# Supplementary figures and images for: How High-Risk Comorbidities Co-Occur in Readmitted Patients With Hip Fracture: Big Data Visual Analytical Approach
Source: JMIR Med Inform. 2020 Oct 26;8(10):e13567. doi: 10.2196/13567 (PMC7652691; doi:10.2196/13567)

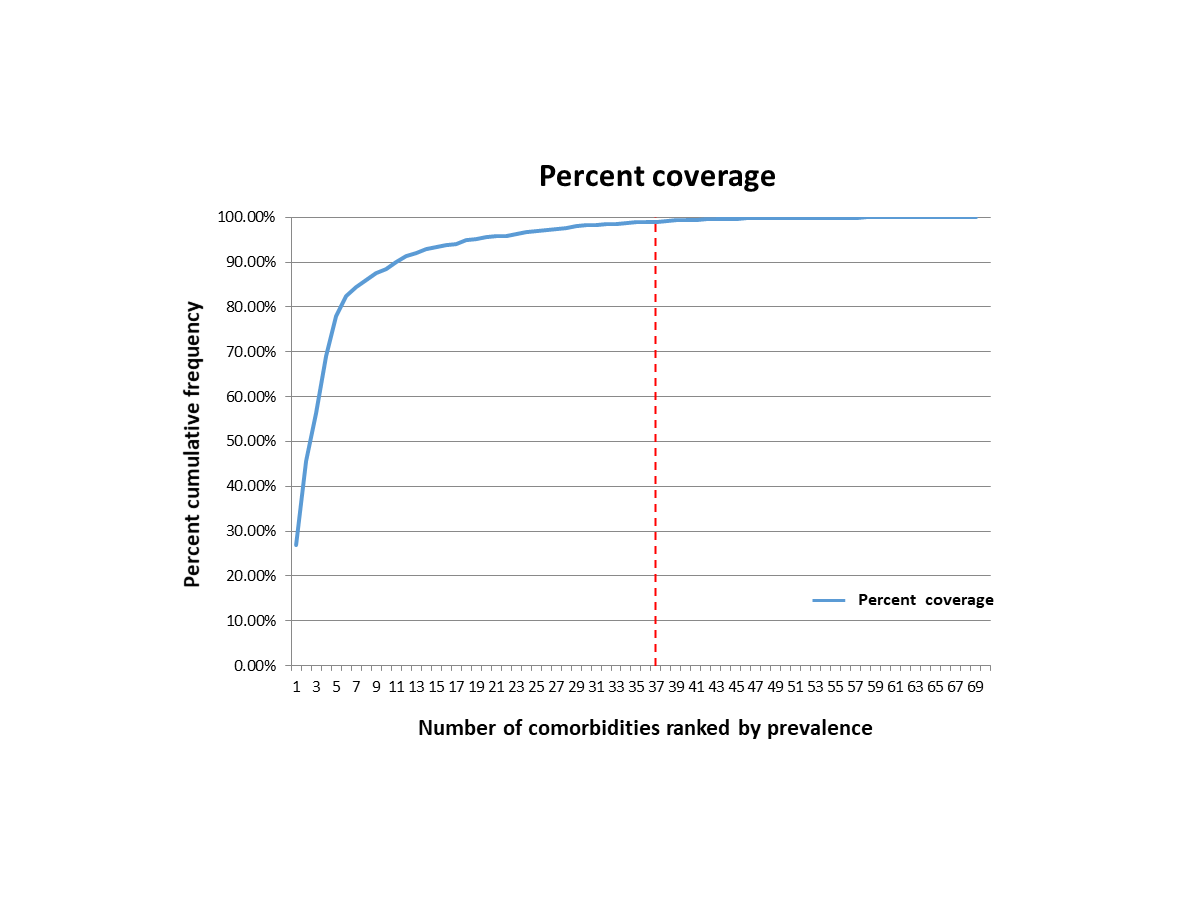

Supplement: Multimedia Appendix 3 [file medinform_v8i10e13567_app3.PNG]
