# Supplementary material for: How High-Risk Comorbidities Co-Occur in Readmitted Patients With Hip Fracture: Big Data Visual Analytical Approach
Source: JMIR Med Inform. 2020 Oct 26;8(10):e13567. doi: 10.2196/13567 (PMC7652691; doi:10.2196/13567)
Supplement: Multimedia Appendix 4 [file medinform_v8i10e13567_app4.docx]

|  |  |  |  |
| --- | --- | --- | --- |
|  | **R packages** | **R functions** |  |
|  | utils | Combn |  |
|  | stats | p.adjust |  |
|  | reshape2 | Melt |  |
|  | igraph | graph.incidence |  |
|  |  | is.connected |  |
|  |  | layout.fruchterman.reingold |  |
|  | fossil | rand.index |  |
|  |  |  |  |
